# Mapping the genetic landscape of early-onset Alzheimer’s disease in a cohort of 36 families

**DOI:** 10.1186/s13195-022-01018-3

**Published:** 2022-06-01

**Authors:** Merel O. Mol, Sven J. van der Lee, Marc Hulsman, Yolande A. L. Pijnenburg, Phillip Scheltens, Netherlands Brain Bank, Harro Seelaar, John C. van Swieten, Laura Donker Kaat, Henne Holstege, Jeroen G. J. van Rooij

**Affiliations:** 1 Alzheimer Center Erasmus MC, Department of Neurology, Erasmus University Medical Center, Rotterdam, The Netherlands; 2 Alzheimer Center Amsterdam, Neurology, Vrije Universiteit Amsterdam, Amsterdam UMC location VUmc, Amsterdam, The Netherlands; 3 Genomics of Neurodegenerative Diseases and Aging, Human Genetics, Vrije Universiteit Amsterdam, Amsterdam UMC location VUmc, Amsterdam, The Netherlands; 4 Amsterdam Neuroscience, Neurodegeneration, Amsterdam, The Netherlands; 5 Delft Bioinformatics Lab, Delft University of Technology, Delft, The Netherlands; 6 Netherlands Institute for Neuroscience, Amsterdam, The Netherlands; 7 Department of Clinical Genetics, Erasmus Medical Center, Rotterdam, The Netherlands

**Keywords:** Alzheimer’s disease, Early-onset Alzheimer disease, Familial Alzheimer disease, Whole exome sequencing, Genetic testing, Genetic counseling, Polygenic inheritance, Polygenic risk score, ApoE

## Abstract

**Background:**

Many families with clinical early-onset Alzheimer’s disease (EOAD) remain genetically unexplained. A combination of genetic factors is not standardly investigated. In addition to monogenic causes, we evaluated the possible polygenic architecture in a large series of families, to assess if genetic testing of familial EOAD could be expanded.

**Methods:**

Thirty-six pedigrees (77 patients) were ascertained from a larger cohort of patients, with relationships determined by genetic data (exome sequencing data and/or SNP arrays). All families included at least one AD patient with symptom onset <70 years. We evaluated segregating rare variants in known dementia-related genes, and other genes or variants if shared by multiple families. *APOE* was genotyped and duplications in *APP* were assessed by targeted test or using SNP array data. We computed polygenic risk scores (PRS) compared with a reference population-based dataset, by imputing SNP arrays or exome sequencing data.

**Results:**

In eight families, we identified a pathogenic variant, including the genes *APP*, *PSEN1*, *SORL1*, and an unexpected *GRN* frameshift variant. *APOE*-ε4 homozygosity was present in eighteen families, showing full segregation with disease in seven families. Eight families harbored a variant of uncertain significance (VUS), of which six included *APOE*-ε4 homozygous carriers. PRS was not higher in the families combined compared with the population mean (beta 0.05, *P* = 0.21), with a maximum increase of 0.61 (OR = 1.84) in the *GRN* family. Subgroup analyses indicated lower PRS in six *APP*/*PSEN1* families compared with the rest (beta −0.22 vs. 0.10; *P* = 0.009) and lower *APOE* burden in all eight families with monogenic cause (beta 0.29 vs. 1.15, *P* = 0.010). Nine families remained without a genetic cause or risk factor identified.

**Conclusion:**

Besides monogenic causes, we suspect a polygenic disease architecture in multiple families based on *APOE* and rare VUS. The risk conveyed by PRS is modest across the studied families. Families without any identified risk factor render suitable candidates for further in-depth genetic evaluation.

**Supplementary Information:**

The online version contains supplementary material available at 10.1186/s13195-022-01018-3.

## Introduction

Alzheimer’s disease (AD) is the leading cause of dementia worldwide [1]. The typical clinical presentation includes progressive memory loss and deficits in other cognitive domains such as orientation, language, and problem solving [2]. In most patients, the first symptoms occur after the age of 65, whereas in ~5% the disease manifests earlier in life. Genes play an important role in the etiology and so far >70 different loci have been associated with AD, implicated in a variety of functional pathways [3, 4]. The heritability of non-Mendelian, late-onset AD (LOAD) is estimated around 60–80% [5] with the ε4 polymorphism in the *APOE* gene as the most common risk factor. A threefold increased AD risk is observed when carrying one *APOE*-ɛ4 allele, and 8-to-12-fold in homozygous carriers [6]. The heritability estimates of early-onset AD (EOAD) are substantially higher (92–100%). There are three known Mendelian AD genes: *APP*, *PSEN1*, and *PSEN2*, which account for 5–10% of EOAD cases [7].

Advances in genetic research techniques and larger sample sizes have enabled the discovery of other variants and susceptibility loci [7, 8]. Genome-wide association studies (GWAS) typically expose common variants with considerate population frequency, but relatively low disease penetrance. Large GWAS within the AD field have uncovered both risk (e.g., *BIN1*, *CR1*) and protective associations (e.g., *CLU*, *PICALM*) [4]. In contrast, next-generation sequencing usually reveals rare variants with higher penetrance such as *SORL1* [9] and *TREM2* [10]. Some genes, including *SORL1* and *ABCA7*, are found to harbor both common and rare variants associated with AD risk [11, 12]. All these studies gradually expose the genetic architecture of AD. Still, since detecting rare variants with moderate to large effects is challenging, a large part of the genetic risk remains unexplained.

Previous research demonstrated that studying families with a high burden of disease provides the opportunity to identify novel variants [13–15]. In addition, the search for risk genes currently includes polygenic risk score (PRS) approaches, a calculation based on the number of risk alleles carried by an individual and the corresponding effect sizes as defined by GWAS. The PRS in AD was previously found to be most elevated in sporadic early-onset cases [16]. In family-based studies, PRS has not been applied to a large extent. Here, we reconstructed 36 small pedigrees consisting of EOAD patients. We have analyzed exome sequencing data of these families to search for rare segregating variants in known and novel genes that might cause disease or act as risk modifiers. In addition, we determined *APOE* genotypes and computed a PRS to investigate the possibility of polygenic etiology underlying AD in these families.

## Methods

### Data collection

**Fig. 1:**
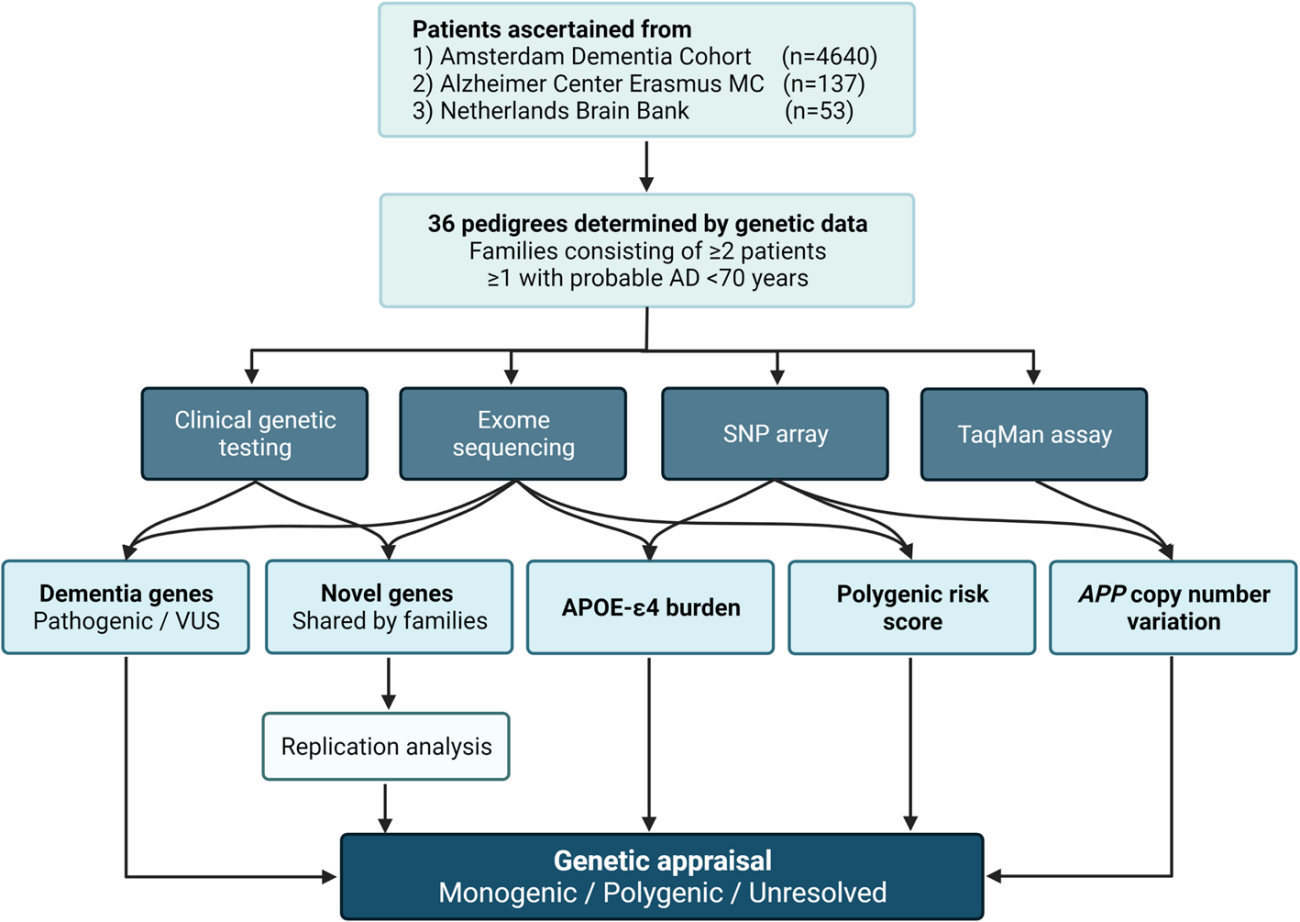
Flowchart of the different analyses performed in this study. Patients with AD were ascertained from two clinical centers (Amsterdam Dementia Cohort and Erasmus MC) and from the Netherlands Brain Bank. Following the reconstruction of 36 pedigrees, various types of genetic data were assessed. Copy number variation in *APP* was tested using SNP array data or by targeted TaqMan assay. Prioritized candidate variants in novel genes were further investigated by replication in a larger cohort of AD patients. *APOE* genotypes and polygenic risk scores were computed for all individuals based on either (imputed) exome sequences or SNP arrays. Finally, all genetic data were reviewed to evaluate the genetic etiology of the families. *Abbreviations: APOE* apolipoprotein E, *APP* amyloid precursor protein, *SNP* single nucleotide polymorphism, *VUS* variant of uncertain significance

A schematic overview of our analysis setup is presented in Fig. 1. Pedigrees were determined based on genetic data of patients ascertained from three different cohorts: (1) 4640 patients from the Amsterdam Dementia Cohort (ADC) [17] with a variety of clinical diagnoses within the dementia spectrum (including AD, mild cognitive impairment [MCI], frontotemporal dementia [FTD], and dementia with Lewy bodies [DLB]); (2) 137 clinical AD patients from Erasmus Medical Center (Erasmus MC); and (3) 53 patients with positive family history and pathologically confirmed AD selected from the Netherlands Brain Bank (NBB). Families consisting of ≥2 patients were selected, including at least one with probable AD [2] and onset <70 years of age. As opposed to the standardly used threshold of 65, we slightly released this age restriction to enable evaluation of patients and families on the border of EOAD and LOAD, hypothetically of interest regarding oligogenic or polygenic nature of disease. Family members aged ≥75 without cognitive complaints were included, if available, to enable segregation analysis of genetic variants. These relatives were confirmed cognitively healthy by MMSE. We reviewed the clinical data of all patients, including the results of previous clinical genetic testing of the patient and/or relatives.

### Genetic data generation and processing

For the majority of patients, single nucleotide polymorphism (SNP) arrays were available as part of the European Alzheimer’s and Dementia Biobank (EADB). Details on data generation and processing have been described previously [3]. Exome sequencing data were generated for all selected patients and eight unaffected relatives. Genomic DNA was extracted from whole blood or frozen post-mortem brain tissue using standard laboratory procedures. DNA samples were paired-end sequenced using Illumina sequencers, after capturing using either Nimblegen SeqCap EZ v3 (ADC and NBB) or v2 (Erasmus MC) capture kits. Raw sequencing data from all sites were collected on a single site and processed using a uniform pipeline as reported recently [18]. In brief, sequenced reads were processed using the Burrows-Wheeler Aligner (BWA) Tool, Picard, and Samtools, and GATK was used for variant calling and quality control according to best practices [19, 20]. All samples were jointly genotyped into a single dataset VCF file (variant call format). Subsequently, a family-VCF was generated for each family. Population database frequencies and functional and impact scores were annotated to variants using ANNOVAR [21].

### Determine pedigrees by genetic data

We assessed family relationships using two independent methods. First, available SNP arrays were pooled for an identical-by-descent (IBD) segment-based method to classify degrees of relatedness using PLINK 1.9 [22]. Second, using the exome sequencing data of all three cohorts, we calculated a kinship coefficient between all pairs of samples using the dataset-VCF file (VCFtools --relatedness2) with common variants only (allele frequency >5%), based on the method of Manichaikul et al. [23]. Previous studies demonstrated that the algorithm reliably infers up to third-degree relationships. Both methods were compared for possible discrepancies and were fully concordant. All identified kinships were checked and confirmed with previously clinically determined pedigrees, when available.

### APOE genotyping and APP copy number analysis

*APOE* genotypes were determined by either (1) clinical testing, (2) sequencing data, or (3) SNP arrays. Additionally, copy number variation (CNV) of the *APP* gene was assessed in at least one member of the included families, either using a made-to-order TaqMan assay (Hs01547105_cn, Applied Biosystems) or by CNV analysis of SNP arrays using the PennCNV algorithm [24].

### Exome sequencing variant filtering

Family-level VCFs were created per identified family, consisting of at least two affected individuals. All variants were filtered based on the following criteria: (1) QD score (quality by depth) of ≥ 5; (2) affecting coding (missense, nonsense, frameshift) or splicing regions (up to 2 bp of exon-intron junctions); (3) minor allele frequency (MAF) < 0.1% in the Genome Aggregation Database (GnomAD) [25]; and (4) heterozygous in all affected individuals and homozygous reference in all unaffected individuals, when available.

#### Dementia-related genes

Following the initial filtering steps, all variants in genes included in a comprehensive list of reported AD and other dementia-related genes/loci (Additional file 1: Table S1) were manually evaluated. In case of other neurodegenerative disorders reported in the family history, related genes were additionally examined. This included assessment of variant type and location, in silico prediction scores, presence in online genetic databases (Alzforum, HEX [healthy exomes], HGMD, LOVD, and ClinVar), and existing literature on the variant or a different variant in the same position. Subsequently, the variants were classified according to guidelines by ACMG (American College of Medical Genetics and Genomics) [26]. Variants predicted to be tolerable by two out of three prediction tools (SIFT, PolyPhen2, MutationTaster) and/or a CADD score <10 were classified as likely benign. Definite pathogenic variants according to genetic databases were confirmed by Sanger sequencing (Applied Biosystems, CA, USA). These families were excluded from subsequent analyses for novel genes.

#### Novel genes

For other genes (not dementia related), we prioritized identical variants occurring in ≥2 families OR different variants in the same gene in ≥3 families, excluding the families with confirmed monogenic cause. Different variants in the same gene shared by only two families were filtered by (1) CADD score >15 in both families and (2) a possible link to dementia/neurodegeneration based on the literature, as per consensus by two researchers. Finally, variants in regions known to give rise to false positives (e.g., low-quality regions, repeats, homologous regions) were manually reviewed using the Integrative Genomics Viewer (IGV) and excluded by expert bioinformaticians when appropriate (Additional file 1: Table S2) [27].

### Replication of candidate variants

To replicate the association of our candidate variants with AD, we used exome sequencing data available from Dutch studies contributing to the Alzheimer Disease European Sequencing (ADES) consortium (ADC, Erasmus MC, NBB, Rotterdam Study, 100-plus study) and from the Amsterdam Human Genetics department (parents of trios and non-dementia cases) consisting of 833 EOAD patients, 521 LOAD patients, and 6949 healthy controls. The set of samples was pruned such that no family relations (up to third degree) remained. Only variants with a MAF < 0.1% were selected and results were categorized based on CADD score (CADD 15, 20, 25, and 30). Population structure was corrected for with 10 PCA components. Only genes with ≥10 carriers were considered for subsequent analysis. Quality control and burden tests were performed using ordinal logistic regression with an EOAD > LOAD > controls labeling as recently described [18]. This method exploits the assumption that the genetic risk will be enriched towards EOAD patients, as can be expected for the candidate variants and genes in this study. Variant-specific analyses were performed with the same approach (minimum of 5 carriers). *P*-values were adjusted for multiple testing using the FDR approach (Benjamini-Hochberg procedure), with FDR < 0.05 considered as suggestively associated with AD.

### Polygenic risk scores

We calculated weighted polygenic risk scores (PRS) for all patients based on genetic variants (Additional file 1: Table S3) showing genome-wide significant association with AD in a recent meta-GWAS by de Rojas et al. [28]. Effect sizes of the variants were obtained from previous GWAS [29–31]. PRS were generated by multiplying the genotype dosage of each risk allele by its respective weight and then summing across all variants. For 56/77 patients included in this study, SNP arrays were available as part of EADB and used to directly genotype variants or impute with high quality (imputation score *R*^2^ > 0.6). For the other 21 patients, we imputed exome sequencing data using the HRC reference panel, including the samples with SNP array data to improve imputation quality. Four out of the 39 variants could not be imputed with sufficient quality (*R*^2^ < 0.3), leaving 35 variants for PRS calculation (median *R*^2^ = 0.73) based on WES data. To enable comparison of all patients, PRS based on SNP arrays were computed using the same 35 variants. Both PRS sets showed a linear correlation (*R*^2^ = 0.61). We scaled the PRS of all individuals by subtracting the mean PRS of an in-house population-based control dataset (*n* = 980); thus, all given effect sizes are relative to the population mean. PRS for each family was obtained by averaging the effect sizes of the affected individuals. We compared PRS between groups of families using an unpaired *t*-test.

### APOE burden

To separate the effects of *APOE* from other genetic variants, we excluded the *APOE* SNPs from the PRS calculation. However, we estimated the risk based on *APOE* for all individuals using the SNP effect sizes from the same meta-GWAS (i.e., −1.20 for rs429358-T-C and 0.47 for rs7412-C-T) [28], scaled to the mean of the same population-based control dataset, to obtain risk estimates on the same scale as PRS. To compare the *APOE* burden across subsets of families, we performed a Mann-Whitney *U* test with continuity on the family-averaged risk. Correlation between APOE-risk and PRS was assessed by Spearman’s rank-sum test.

### Genetic findings categorized based on clinical actionability

We evaluated the impact of the different genetic components for each family. The cumulative genetic evidence and potential relationships between variant carrier status (pathogenic variant or a VUS), *APOE* burden, and PRS were assessed, leading to categorization of the families as follows: (1) genetic cause identified, sufficient to be reported back to patients (i.e., passing actionable risk threshold); (2) one or more genetic risk factors identified suggesting potential polygenic etiology (i.e., passing genetic risk threshold, but insufficient for clinical action as the complete cause remains uncertain); (3) no genetic risk factors identified after evaluation of variants in dementia-related genes, *APOE*, and PRS, suggesting the presence of additional, yet undiscovered genetic risk factors.

## Results

### Clinical demographics of included families

With the pooled genetic data of 4840 patients, we constructed 36 families (at least second-degree relation) comprising at least two patients (*n* = 77). Seven pedigrees were previously clinically established and used to validate our two methods for determining relatedness. Both approaches were able to define first- and second-degree relationships and were fully concordant.

For each family, clinical and genetic findings are summarized in Table 1. All included families consisted of at least one patient with (probable) AD before the age of 70. The average age at onset of all affected individuals was 63 years. A lumbar puncture was performed in 32/36 probands, confirming decreased concentrations of β-amyloid in cerebrospinal fluid (CSF). Occasionally, relatives had been diagnosed with other phenotypes on the dementia spectrum (MCI, FTD). Four families included an unaffected relative aged >75. Detailed information on the individual level is provided in Additional file 1 (Table S4).

**Table 1:** Summary of the clinical characteristics and genetic findings of the 36 families evaluated in this study

Family ID	Patients (*n*)	Age at onset (average)	Clinical diagnoses	Family history - other relatives	PV	VUS	APOE-risk*	PRS*	Summary (genetics)
1	2	57.0	AD	Unknown	*APP*		0.24	−0.49	PV
2	2	48.0	AD	Dementia NOS	*GRN*		0.24	0.61	PV, PRS
3	2	69.5	AD	EOAD	*PSEN1*		0.01	−0.26	PV
4	2	56.0	AD	EOAD	*PSEN1*		−0.36	−0.08	PV
5	2	64.5	AD	EOAD	*PSEN1*		−0.36	−0.33	PV
6	2	49.0	AD	EOAD	*PSEN1*		0.84	0.06	PV
7	2	59.0	AD	EOAD	*PSEN1*		0.24	−0.23	PV
8	2	57.5	AD	EOAD	*SORL1*		1.44	0.47	PV, APOE, PRS
9	2	65.0	AD, MCI	EOAD		*ABCA7*	2.04	0.20	VUS, APOE
10	2	64.0	AD	Dementia NOS		*BIN1*	1.44	0.02	VUS, APOE
11	2	69.5	AD, MCI	Dementia NOS		*CR1*	1.44	0.37	VUS, APOE
12	2	69.5	AD	EOAD		*FERMT2*	2.04	0.20	VUS, APOE
13	2	69.0	AD	Dementia NOS, PD		*MADD*	2.04	−0.02	VUS, APOE
14	2	63.0	AD	Dementia NOS		*PRNP*	0.24	0.12	VUS
15	2	59.5	AD	EOAD		*SORL1*	1.44	0.15	VUS, APOE
16	2	73.0	AD	EOAD		*SQSTM1*	0.84	0.42	VUS
17	2	68.0	AD	Dementia NOS			1.44	0.60	APOE, PRS
18	4	70.3	AD	Dementia NOS			2.04	0.56	APOE, PRS
19	2	62.5	AD, FTD	Dementia NOS			1.44	−0.17	APOE
20	2	68.5	AD	ALS			1.44	−0.14	APOE
21	2	62.0	AD	Dementia NOS, PSY			2.04	0.32	APOE
22	2	56.0	AD, MCI	AD, FTD			2.04	−0.15	APOE
23	2	65.0	AD	Dementia NOS			1.44	−0.04	APOE
24	2	68.0	AD, MCI	LOAD			1.44	−0.08	APOE
25	3	56.7	AD, CAA, MCI	EOAD			1.64	0.41	APOE
26	3	59.0	AD, MCI	EOAD			1.24	−0.30	APOE
27	2	59.0	AD, FTD	Dementia NOS			2.04	0.01	APOE
28	2	67.5	AD	EOAD, PD			−0.36	−0.18	Risk factors unknown
29	3	63.5	AD	Dementia NOS			0.44	−0.33	Risk factors unknown
30	2	64.9	AD	Unknown			−0.36	−0.10	Risk factors unknown
31	2	73.0	AD	Dementia NOS			0.84	−0.01	Risk factors unknown
32	2	63.5	AD, MCI	Dementia NOS			−0.36	−0.19	Risk factors unknown
33	2	63.5	AD	LOAD			0.84	0.19	Risk factors unknown
34	2	59.5	AD, MCI	Dementia NOS, PSY			0.24	0.04	Risk factors unknown
35	2	69.0	AD	Dementia NOS			0.24	−0.15	Risk factors unknown
36	2	59.0	AD	Dementia NOS			0.84	0.19	Risk factors unknown
Total	**77**								
Average		**63.1**					**0.96**	**3.50**	

In four families (12, 18, 19, and 30), an unaffected relative was included for segregation analysis. Data on these individuals, as well as all individual patients, are provided in Table S3. *APOE-risk and PRS indicate the increased AD risk for each family based on *APOE* and PRS effect sizes, respectively, after scaling to the mean of an in-house population dataset (*n* = 980). *Abbreviations: ALS*, amyotrophic lateral sclerosis; *AD*, Alzheimer’s disease; *CAA*, cerebral amyloid angiopathy; *Dementia NOS*, dementia not otherwise specified; *EOAD*, early-onset AD; *FTD*, frontotemporal dementia; *LOAD*, late-onset AD; *MCI*, mild cognitive impairment; *PD*, Parkinson’s disease; *PSY*, psychiatric disorder; *PV*, pathogenic variant; *VUS*, variant of uncertain significance

### Pathogenic variants in dementia-related genes

In eight families, we detected six different pathogenic variants in known dementia genes (Table 2). Six families harbored known causal pathogenic variants in *PSEN1* and *APP*, with pathological confirmation of AD in two patients*.* The identified frameshift deletion in *SORL1* (p.P961fs), absent in GnomAD, has not yet been reported in variant databases. We classified this variant as pathogenic, as previous studies show that truncating *SORL1* variants are highly penetrant [32]. This is consistent with the high burden of EOAD in this family.

**Table 2:** Pathogenic variants and rare VUS identified in dementia-related genes

Gene	Transcript	Nucleotide change	Amino acid change	MAF GnomAD Total	MAF GnomAD NFE	CADD (v1.3)
**Pathogenic variants**						
*APP*^a^	NM_000484	2149G>A	V717I	0	0	34
*GRN*	NM_002087	388_391delCAGT	Q130SfsX125	7.0^e^−06	1.5^e^−05	NA
*PSEN1*^b^	NM_000021	786G>C	L262F	0	0	25.6
*PSEN1*	NM_000021	692C>T	A231V	0	0	34
*PSEN1*^a,b^	NM_000021	236C>T	A79V	1.4^e^−05	2.3^e^−05	33
*SORL1*	NM_003105	2882_2885delCGCA	P961fs	0	0	NA
**Variants of uncertain significance**						
*ABCA7*	NM_019112	3698G>A	R1233H	2.3^e^−05	4.1^e^−05	23.9
*BIN1*	NM_004305	746C>T	T249M	2.6^e^−05	3.2^e^−05	24.6
*CR1*	NM_000573	4162G>A	E1388K	0	0	24
*FERMT2*	NM_006832	667G>A	V223I	1.0^e^−04	2.1^e^−04	18.8
*MADD*	NM_003682	2308dupA	D769fs	2.0^e^−04	4.3^e^−04	NA
*PRNP*	NM_000311	130C>T	P44S	1.8^e^−05	0	23.4
*SORL1*^c^	NM_003105	4576G>T	A1526S	0	0	18.2
*SQSTM1*	NM_003900	c.420C>A	Y140X	0	0	35

^a^Pathological confirmation of AD in one affected patient carrying this variant. ^b^Detected in two families. ^c^Confirmed by Sanger sequencing and reported previously by Holstege et al., *EJHG* (2017). *Abbreviations: MAF*, minor allele frequency; *NFE*, non-Finnish European population

In one family, we identified a pathogenic variant in the gene *GRN*, known to cause FTD. The frameshift variant (p.Q130SfsX125) was among the first *GRN* variants reported in FTD families and confirmed pathogenic in later studies [33–35]. The family in the current study consisted of two siblings with remarkably early onset (<50 years) of typical AD, without symptoms suggestive of FTD. The diagnosis of AD was supported by CSF biomarkers in both siblings (Aβ 674/477, t-tau 649/573, p-tau 78/78). Plasma GRN levels were not available for these patients. MRI showed generalized atrophy with relative sparing of the hippocampi. Family history was positive for EOAD in first- and second-degree relatives.

### APOE burden

The majority of the families included *APOE*-ε4 heterozygous patients (24/36 families; 67%), and at least one homozygous carrier was found in 18 families (18/36; 50%). Of these, seven families showed complete segregation of *APOE*-ε4 homozygosity. Only one patient carried an *APOE*-ε2 allele. Of the families with a pathogenic variant, only the family with the *SORL1* frameshift also included an *APOE*-ε4 homozygous carrier.

We evaluated the *APOE* burden among the families using previously computed effect sizes for each genotype, scaled to the mean of our population-based control dataset (*n* = 980). As such, a risk of 0 indicates the population mean (OR = 1), whereas an effect size of 1, for instance, implies an increased risk with odds ratio 2.7. We observed a significantly smaller risk conveyed by *APOE* in the eight families with pathogenic variants as compared with the rest (0.29 vs. 1.15, *P* = 0.010) (Table 3). To evaluate the presence of additional genetic causes or risk factors (e.g., rare variants or polygenic risk), we kept the families with high *APOE* burden — and without monogenic cause — in subsequent analyses.

**Table 3:** Subgroup analyses of AD risk in the families imposed by *APOE* and polygenic risk score

	Total	Pathogenic variant		No pathogenic variant		
		All	***APP***/***PSEN1***	VUS	Low APOE-risk (<1)	High APOE-risk (>2)
APOE-risk						
No. of families	36	8	6	8	NA	NA
Mean	0.96	0.29	0.10	1.44	NA	NA
Standard deviation	0.83	0.60	0.45	0.64	NA	NA
vs. Rest, *P*-value	NA	0.010*	0.006*	0.063	NA	NA
Polygenic risk score						
No. of families	36	8	6	8	11	7
Mean	0.05	0.03	−0.22	0.18	−0.001	0.16
Standard deviation	0.28	0.39	0.19	0.15	0.22	0.24
vs. Rest, *P*-value	NA	0.379	0.009*	0.129	0.515	0.241

The increased risk conveyed by *APOE* and PRS was compared across subsets of families with/without pathogenic variants (all eight or only those with *APP*/*PSEN1* variants), with/without a VUS, and with/without increased APOE-risk. Low APOE-risk (<1, OR = 2.7) was defined by the absence of *APOE*-ε44 carriers in the family, whereas a high risk (>2, OR = 7.34) indicates complete segregation with *APOE*-ε44. For APOE-risk, families were compared using a Mann-Whitney *U* test, whereas for PRS an unpaired *t*-test was performed. *Below significance threshold of *P* < 0.05. *Abbreviations: VUS* variant of uncertain significance

### Variants of uncertain significance in dementia-related genes

In a broad panel of dementia-related genes, we identified 10 rare segregating variants. Two missense variants in *CR1* and *GRN* were classified as likely benign based on in silico predictions, leaving eight variants of uncertain significance (VUS), including missense variants in the AD risk genes *SORL1*, *ABCA7*, and *FERMT2* (Table 2). In all eight families, at least one patient was *APOE*-ε4 heterozygous, and six families included *APOE*-ε4 homozygous carriers. The difference in APOE-risk between families with and without a VUS was not statistically significant (1.44 vs. 0.82; *P* = 0.063).

We identified a nonsense variant in *SQSTM1* (p.Y140X), a gene associated with FTD and amyotrophic lateral sclerosis (ALS) [36]. The variant is absent from all genetic databases and leads to a premature stop codon removing the last 301 amino acids, resulting in a strong deleteriousness prediction (CADD 35), although *SQSTM1* seems relatively tolerant to loss of function (pLI 0.001) [25]. The two patients carrying this variant both had CSF profiles consistent with AD. Both were *APOE*-ε4 heterozygous. The family history indicated early death of the father, but five out of 11 of his siblings had suffered from early-onset dementia. Their clinical presentations did not include symptoms suggestive of FTD/ALS. Unfortunately, no additional relatives were currently available for further segregation analysis.

We also found a variant in the gene *PRNP*, which is associated with familial forms of prion disease. The clinical picture of this family did not encompass atypical features reminiscent of inherited prion disease such as Creutzfeldt-Jakob disease.

### Other neurodegenerative disease genes

Besides dementia, some patients reported other neurodegenerative disorders in the family history. In two families, relatives had been diagnosed with Parkinson’s disease (PD). We screened the sequencing data of these families for PD-related genes [37], but did not detect any candidate variants. Similarly, we screened four families with FTD and/or ALS phenotypes for genetic variants in ALS-related genes [38], which did not yield any result. A repeat expansion in *C9orf72* was excluded in three out of four families. For the remaining family, the CSF profile was consistent with the diagnosis of AD, lowering the possibility of FTD-*C9orf72*.

### Variants in novel genes

A total of 28 families without definite pathogenic variant (including those with a VUS and high *APOE*-ε4 burden) were included for further analyses, to identify variants in novel genes possibly associated with AD risk. On average, 76 variants were remaining in each of these families after initial filtering steps (coding, segregating variants with MAF < 0.1%). Genes were prioritized based on two different approaches. First, we ascertained shared variants by ≥2 families and different variants in one gene in ≥3 families, summing up to 42 unique genes. After excluding likely false positive variants (Additional file 1: Table S2), 28 genes remained for replication analysis. In parallel, we evaluated different variants in genes shared by two families with CADD > 15, further prioritized by possible association with neurodegeneration based on literature. This resulted in the selection of nine additional genes (further details in Additional file 1: Table S5). Following the two parallel strategies, we included 37 genes (78 variants) in the replication analysis. A complete list of all variants is provided in Additional file 1 (Table S6).

### Replication analysis

For our prioritized set of genes, we performed a genetic association test on both gene and variant levels, using exome sequencing data from Dutch studies contributing to the ADES consortium. None of the genes or variants tested was significantly associated with EOAD nor LOAD in this dataset as compared with controls (Tables S7-8), although 40/78 very rare variants could not be tested due to limited power. The most significant association was found for *LRP1B* in patients carrying variants with CADD>15 (OR = 1.13, unadjusted *P* = 0.08). The largest effect was found for variants with CADD>25 in the gene *RBFOX1* (OR = 2.14, unadjusted *P* = 0.15). In the per-variant analysis, a missense variant in *FNDC1* was present more often in cases compared with controls with nominal significance (OR = 3.12, unadjusted *P* = 0.03).

### Polygenic risk score

**Fig. 2:**
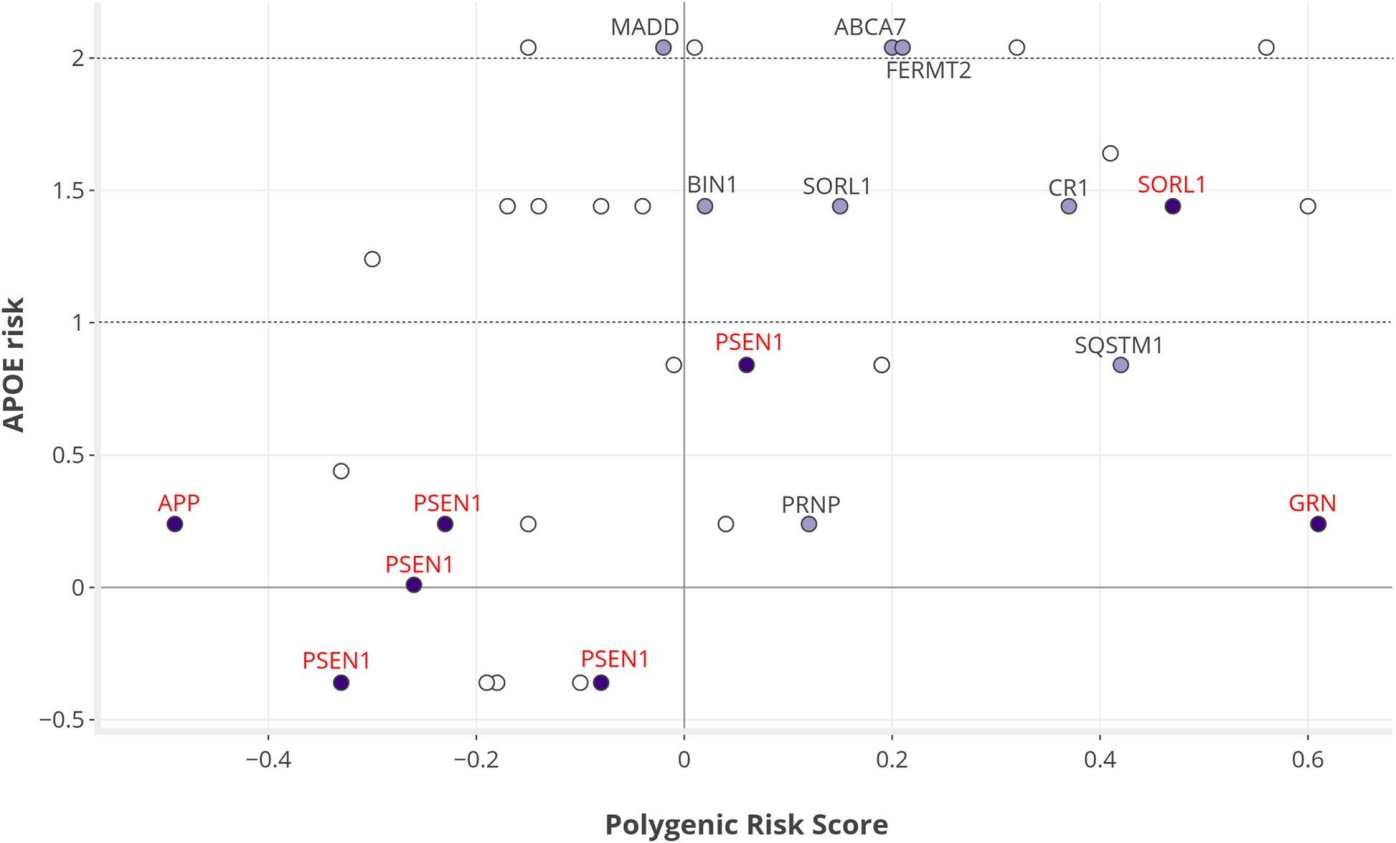
The AD risk based on *APOE* and polygenic risk score (PRS) in the 36 families. Each node represents one family, with PRS plotted on the *x*-axis and APOE-risk on the *y*-axis. The effect sizes of PRS and *APOE* were scaled to the mean of an in-house population-based control dataset (*n* = 980). For interpretation of the effect sizes: 0, OR = 1; 1, OR = 2.7; and 2, OR = 7.4. The horizontal lines for APOE-risk indicate families with risk >1 (i.e., at least one *APOE*-ε44 carrier) and risk >2 (i.e., fully segregating with *APOE*-ε44). Dark colored nodes depict families with pathogenic variant (*n *= 8), whereas light colored nodes represent families with a variant of uncertain significance (*n* = 8). Families with a pathogenic variant are clustered in the lower left corner, with a minor impact of *APOE* and PRS. Other families in the same region, yet without monogenic defect, would be suitable candidates for further genetic evaluation. Families with a relatively high *APOE* burden are located in the upper part of the plot, which also includes most families with a VUS. The overall contribution of PRS seems modest (average beta = 0.05, OR = 1.05), with the highest risk observed in the family with a *GRN* variant (beta = 0.61, OR = 1.84)

Similar to *APOE*, PRS of the families were scaled to the mean of our population-based control dataset. Figure 2 depicts the increased risks based on PRS and *APOE* for each family, highlighting families with pathogenic variants and a VUS. An overview of subgroup comparisons with statistical output is provided in Table 3. On average, the polygenic risk of the AD families was 0.05 higher (OR = 1.05) than the population mean (SD 0.28, *P* = 0.206) and did not correlate with age at onset (*R*^2^ < 0.01). PRS of the eight families with a pathogenic variant did not deviate from the rest (−0.03 vs 0.07; *P* = 0.379). However, the six families with *APP* or *PSEN1* variants showed significantly lower PRS (−0.22 vs. 0.10; *P* = 0.009), whereas those with a pathogenic variant in *SORL1* and *GRN* had higher scores (0.47 and 0.61, respectively).

When selecting 11 families without monogenic cause and low APOE-risk (<1), PRS did not differ from the other 25 families (−0.001 vs. 0.07, *P* = 0.515), nor was a significant difference observed when comparing families with and without a VUS (0.18 vs. 0.01; *P* = 0.129). When excluding the families with pathogenic variant, a positive correlation was observed between PRS and APOE-risk (*R*_s_ = 0.48, *P* = 0.003) in the remaining 28 families.

### Families categorized according to genetic findings

**Fig. 3:**
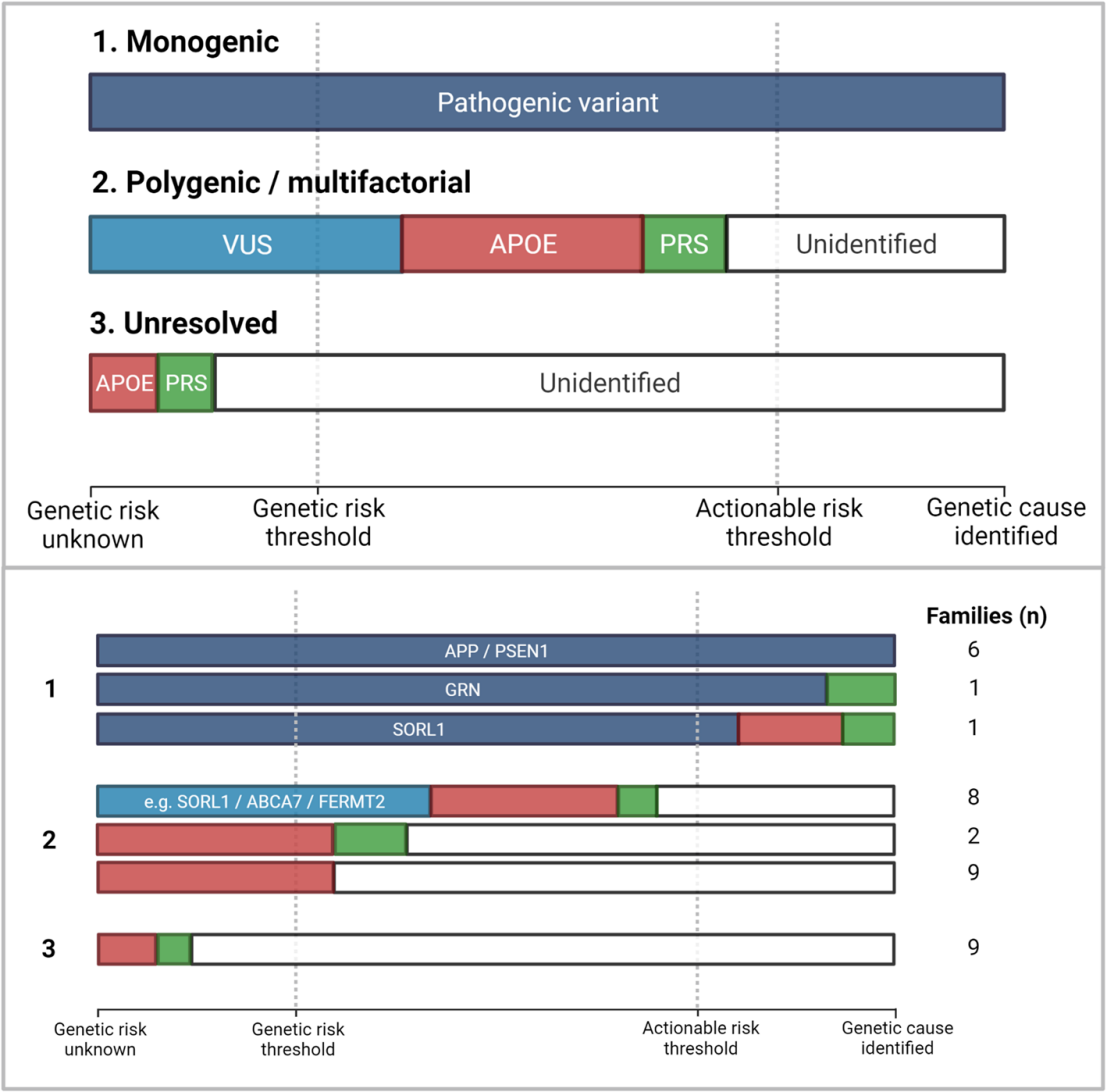
Schematic summary of the genetic etiology of the families, categorized into three groups. We classified the families into three distinct groups based on the clinical actionability of the genetic findings, as illustrated systematically in the upper panel and for the 36 families in the lower panel. The first group represents families with a pathogenic variant identified as the cause of disease, passing a clinically actionable threshold where results could be returned to the patients and relatives. The second category includes families with (possible) genetic risk factors (*APOE*, VUS, and PRS). These are not clinically actionable (i.e., these families are clearly burdened by genetic risk factors, but the complete cause is uncertain). Possibly, additional components of the genetic risk remain unidentified. The last group comprises families without any genetic risk factor currently identified, despite early onset and positive family history. The lower panel shows that we can genetically explain eight families, suspect a partially identified polygenic/multifactorial etiology in 19 families, whereas nine remain completely unresolved

Following the assessment of dementia-related genes, *APOE* genotypes, and PRS, we classified the families into three distinct categories as schematically visualized in Fig. 3. The first represents eight families with a monogenic cause identified, meriting disclosure to patients and relatives. The second group comprises 19 families in whom we found one or more genetic risk factors, suggesting polygenic or multifactorial etiology. As these factors do not sufficiently explain the disease occurrence, clinical reporting or further action is currently not indicated in these families. The last group contains nine families without any identified genetic cause or risk factor, despite the early onset of AD and the positive family history, in which additional genetic factors may be uncovered.

## Discussion

In this genetic study of AD, we analyzed exome sequencing data of 36 small families with a high burden of early-onset disease. In addition to known monogenic causes, we evaluated rare novel variants as well as the increased genetic risk based on *APOE* and polygenic risk profiling.

### The genetic landscape of EOAD includes FTD-related genes

Besides pathogenic variants in *APP*, *PSEN1*, and *SORL1*, we discovered a deleterious *GRN* frameshift variant (p.Q130SfsX125) in one family. The variant is known to cause FTD [33, 39] and has not been associated with clinical AD, although one family was described with profound AD neuropathology (reported as UBC11, Braak stage VI) [40]. Although FTD-*GRN* patients may show coexistent AD pathology [41], certain *GRN* variants have been suggested as the direct cause of AD [42] through several proposed mechanisms (i.e., Aβ clearance, tau phosphorylation, neuroinflammation), indicating shared pathways between AD and progranulin [43]. As such, we propose that the identified variant — possibly in concert with an AD polygenic risk profile (OR = 1.84) — plays a causal role in this family and should be reported in genetic counseling. It would be of interest to evaluate PRS in a larger cohort of *GRN* carriers presenting with an AD phenotype. In a clinical setting, we recommend to examine an extended panel of dementia genes in familial EOAD, when *APP*, *PSEN1*, and *PSEN2* are tested negative, as was suggested before [44]. Following a family history of PD and ALS, we also screened genes related to these disorders in a few families. As this did not yield any candidate variants, it remains unclear whether such genes should be standardly examined in AD.

The second non-traditional AD finding is a rare stop-gained variant in *SQSTM1*. This gene encodes the scaffold protein p62, involved in diverse signaling pathways associated with neurodegeneration such as autophagy, inflammation, and tau degradation [45]. Several studies have shown that reduced *SQSTM1* gene expression and lack of cytoplasmic p62 provoke the aggregation of pathological tau and neurofibrillary tangles [46, 47], implying an important role of p62 in the pathogenesis of AD. Although no risk or causal *SQSTM1* variants have been identified in AD patients so far [48], the wide clinical spectrum of *SQSTM1* variants is exemplified by case reports of familial hippocampal amnestic syndrome closely resembling AD [49] and atypical FTD with prominent memory decline [50]. Replication of *SQSTM1* variants in larger AD cohorts is needed before it can be included in genetic risk prediction.

Regarding the gene *PRNP*, several variants have been suggested to play a role in other neurodegenerative disorders besides prion disease [51]. For instance, variants in the same N-terminal domain as the here identified variant were previously detected in FTD and AD patients [52, 53]. As such, we cannot exclude a causal role, although currently its classification remains a VUS.

### Evaluating the risk imposed by APOE and rare VUS

We identified a substantial burden of *APOE*, with heterozygous ε4-allele carriers in 67% of the families and homozygosity in 50%. Although the risk conferred by two *APOE*-ε4 alleles can cause semi-dominant inheritance with AD lifetime risk estimates of 30% by 75 years of age [54], we cannot be sure if *APOE* might be the sole genetic factor in these families, or whether some carry additional factors — genetic and/or environmental — to trigger disease initiation.

Following the relatively low burden of *APOE* and PRS in families with a known pathogenic variant, we anticipated a similar low burden in families with a VUS, if these are pathogenic. However, six out of eight families with a VUS showed a high occurrence of *APOE*-ε4 homozygosity. Moreover, we did not observe a difference in PRS across families with and without a VUS. It suggests that the identified VUS are insufficient by themselves to cause disease and that the genetic burden for AD in these families is multifactorial, although individual families might have a high burden of *APOE* or PRS by chance, on top of a monogenic cause.

A possible additive risk of VUS and *APOE* is supported by their observed frequent co-occurrence. In addition, interactions between genetic factors influencing the same biological pathways might point in the direction of a cumulative effect, for example between *APOE* and the identified VUS in *SORL1* and *ABCA7*, which are both involved in lipid metabolic pathways [12, 32]. Previous work has indeed shown that the combination of pathogenic variants in *SORL1* with *APOE*-ε4 increases penetrance [55]. Additional risk genes in which we detected a VUS are also associated with lipid metabolism (e.g., *FERMT2*, *BIN1*, *CR1*) [56, 57], reinforcing the possibility that convergence of these genetic factors with *APOE* on this particular pathway bestows an increased AD risk.

### Modest contribution of PRS in familial EOAD

The overall impact of PRS in the current families was modest, similar to earlier findings in autosomal dominant AD [16]. It should be pointed out that the PRS was constructed based on GWAS data of mostly LOAD patients, but was replicated in EOAD with similar effect estimates [28]. Although an oversimplification of polygenic inheritance, we calculated a family-averaged PRS to represent the shared polygenic burden. This allowed us to compare the contribution of PRS across families, also in conjunction with other genetic factors.

The four families with the highest PRS showed, on average, a risk score of 0.56 (OR = 1.75) as compared with the general population, which is not evidently higher than previously reported effects in LOAD [16, 28], and appears small when set against the impact of *APOE* with maximum risk of 2.04 (OR = 7.34) (Fig. 2). Families with causal variants in *APP* and *PSEN1* showed especially low PRS — similar to the low burden of *APOE* — consistent with the perception that these variants are on their own sufficient to cause AD. This raises the notion whether families with similarly low PRS and *APOE* burden (i.e., nine families as shown in Figs. 2 and 3) may harbor monogenic causes (pathogenic variants) that currently remain undetected. As our analyses investigating rare variants shared by multiple families did not yield potential candidates, we would prioritize these families for genetic evaluation using other methods, as well as attempting to expand specifically these pedigrees to optimize segregation analysis. Investigating multiple affected relatives using whole genome sequencing could aid in the identification of novel genetic defects, including intronic and structural variation.

Similar to *APOE* and rare VUS, a cumulative effect might apply to the risk conveyed by *APOE* and PRS, as we observed a positive correlation between the two. Future work is needed to clarify if a combined signal might be based on biological interactions, endorsed by a recent study demonstrating a disproportionally large effect of PRS (without *APOE*) among *APOE*-ε4 carriers with earlier disease onset [58].

### Genetic testing of APOE and PRS in clinical practice

*APOE* genotyping is not incorporated in current risk predictions, because it is neither diagnostic nor sufficiently predictive, and at present has no clinical consequences [59]. These guidelines will likely not change until effective treatments become available. Nevertheless, given the fact that half of the families studied here were affected by *APOE*-ε4 homozygosity, in some instances accompanied by a potentially relevant VUS, a pertinent question is at which point the risk is sufficiently high to include these factors in genetic risk modeling and counseling. Importantly, *APOE* genotyping is currently used in clinical trials and is available via direct-to-consumer genetic testing [60]. Previous studies suggest that genetic risk disclosure of *APOE* can be safely performed in a clinical setting, provided that appropriate and standardized counseling protocols are applied [61, 62]. On the other hand, the risk of misunderstanding or overinterpretation is an important issue, which must be carefully weighed against the possible personal utility of disclosure for the individual patient [63]. At any rate, we urge *APOE* genotyping in a research setting to further clarify its impact and interplay with other (genetic) factors.

Although it might exert a contributory effect in some families, at present, we do not recommend PRS as a diagnostic tool in familial EOAD as studied here, following its relatively small impact in all families. Although a few families showed higher risk, this currently seems insufficient to justify clinical action — similar to *APOE* — until disease-modifying therapies or cost-effective preventive measures become available. As a result, although the PRS will likely improve when more variants are added to it, we foresee it will not change this view on its clinical utility in familial EOAD in the near future.

Currently, PRS seems most applicable to estimate risk in late-onset forms of AD [28, 64] and has proven useful to stratify patients for clinical trials to reduce variability [65, 66], and for genes that show reduced penetrance (e.g., *PSEN2*), PRS might influence clinical expression, as has been shown for other diseases such as hereditary breast cancer [67]. Additional work is required to support these potential applications of PRS in a clinical setting.

### Future directions

By evaluating various genetic factors in parallel, we investigated components of multifactorial genetic risk associated with AD. To validate whether individual genetic factors (pathogenic variants, VUS, *APOE*, PRS) interact and are thus multiplicative or additive must be determined by population studies with sufficient carriers to populate such models. For instance, the variability in disease penetrance and expression of *APOE* might be explained by interactions with other genes involved in the same pathways, as supported by prior studies investigating biological interactions and correlations with clinical measures [68–71]. Large-scale genetic studies could thereby also investigate how the combined genetic signals translate into expected penetrance at a certain age at onset. Our findings in non-traditional AD genes, of which the clinical impact might be modulated by other factors such as PRS, need to be replicated in extended cohorts. We anticipate that this will contribute to the development of more inclusive risk algorithms. Finally, we propose that the commonly used threshold of 65 years for EOAD could be slightly released when evaluating oligogenic/polygenic inheritance, although the most appropriate age remains a topic of investigation.

### Limitations

There are some limitations to this study. First, we prioritized variants shared by two families by employing several strategies (i.e., allele frequency, CADD score, literature search), possibly discarding variants that could be relevant. Second, we tested potential candidates in a sizeable cohort, but the power was limited for the assessment of very rare variants and this dataset was not specifically enriched for cases with a positive family history. Replication of these variants is required in additional case-control samples. Finally, for a subset of patients, we computed PRS from imputed sequencing data with lower imputation quality than SNP arrays. This could have impacted results, despite the high correlation (*R*^2^ = 0.6) between the two datasets.

## Conclusion

Our comprehensive study of familial AD, with each family having at least one patient with age at onset <70, demonstrates its broad genetic framework. Besides monogenic causes, we suspect a polygenic/multifactorial etiology in around half of the families based on *APOE* and rare variants in dementia-related genes. Although PRS may contribute to disease risk, the extent of its effect is small and warrants further investigation before it can be incorporated in clinical risk prediction. Importantly, we showed that assessing *APOE* burden and PRS can help distinguish those patients with a higher a priori probability for a monogenic cause, thus, rendering them suitable candidates for further in-depth genetic evaluation.

## Supplementary Information

**Additional file 1: Table S1**. List of dementia related genes and loci that were analyzed in the exome sequencing data for each family separately. **Table S2**. List of variants and genes that were excluded following manual evaluation using the Integrative Genomics Viewer (IGV). **Table S3**. List of variants (*n *= 35) included to compute the Polygenic Risk Score (PRS). **Table S4**. Clinical and genetic findings of all affected individuals (*n *= 77) and unaffected relatives (*n *= 4). **Table S5**. Selection of genes shared by two families with possible connection to dementia based on literature search. **Table S6**. Total list of candidate genes (*n *= 37) and variants (*n *= 78) occurring in ≥2 families selected for replication analysis. **Table S7**. Replication analysis of candidate genes. **Table S8**. Replication analysis of candidate variants.

## Data Availability

The genetic variants analyzed in this study and summary statistics are included in the Supplementary data files. The full datasets generated and/or analyzed during the current study are not publicly available due to privacy restrictions applicable to genetic data from human subjects.
